# Processing of masked and unmasked emotional faces under different attentional conditions: an electrophysiological investigation

**DOI:** 10.3389/fpsyg.2015.01691

**Published:** 2015-10-31

**Authors:** Marzia Del Zotto, Alan J. Pegna

**Affiliations:** ^1^Laboratory of Experimental Neuropsychology, Faculty of Psychology and Educational Sciences, University of Geneva, Geneva, Switzerland; ^2^School of Psychology, University of Queensland, Brisbane, QLD, Australia

**Keywords:** ERP, emotions, faces, subliminal, masking, awareness, selective attention

## Abstract

In order to investigate the interactions between non-spatial selective attention, awareness and emotion processing, we carried out an ERP study using a backward masking paradigm, in which angry, fearful, happy, and neutral facial expressions were presented, while participants attempted to detect the presence of one or the other category of facial expressions in the different experimental blocks. ERP results showed that negative emotions enhanced an early N170 response over temporal-occipital leads in both masked and unmasked conditions, independently of selective attention. A later effect arising at the P2 was linked to awareness. Finally, selective attention was found to affect the N2 and N3 components over occipito-parietal leads. Our findings reveal that (i) the initial processing of facial expressions arises prior to attention and awareness; (ii) attention and awareness give rise to temporally distinct periods of activation independently of the type of emotion with only a partial degree of overlap; and (iii) selective attention appears to be influenced by the emotional nature of the stimuli, which in turn impinges on unconscious processing at a very early stage. This study confirms previous reports that negative facial expressions can be processed rapidly, in absence of visual awareness and independently of selective attention. On the other hand, attention and awareness may operate in a synergistic way, depending on task demand.

## Introduction

In the two last decades, many studies have focused on conscious and unconscious processing of emotional stimuli (for reviews, see Lindquist et al., 2012; Pourtois et al., 2013). One of the most extensively investigated categories of stimuli in this field are human facial expressions. In fact, due to their critical role in social, emotional and cognitive function, human faces constitute a biologically relevant category of visual stimuli, thought to be processed very rapidly and leading to an immediate regulation of behavior. Notably, it has been shown that emotions can selectively influence early aspects of visual perception, modulating the strength of the neuronal signal (Batty and Taylor, 2003; Bocanegra and Zeelenberg, 2009).

Along these lines, electrophysiological studies using face stimuli have provided evidence that faces can be processed at an early stage and without awareness both in healthy controls (e.g., Kiss and Eimer, 2008; Pegna et al., 2008, 2011) and in patients with cortical blindness (Gonzalez Andino et al., 2009; Del Zotto et al., 2013). Using a backward masking paradigm in healthy participants, Kiss and Eimer (2008) found that both subliminal and supraliminal fearful faces produced an enhanced early frontal positivity compared to neutral faces between 140 and 180 ms post-stimulus. In addition, the N2 (180–250 ms) was modulated by emotion at frontal and central sites, though only on subliminal trials. In another backward masking study, Pegna et al. (2008) found an increased N170 for masked fearful compared to non-fearful (happy and neutral) faces. Moreover, the N2 was observed to be greater over the right posterior leads for fearful compared to non-fearful expressions, increasing progressively with target detectability. Despite the discrepancies between these two studies regarding the location of the effects (possibly due to the use of different reference electrodes), these observations reveal that negative (fearful) emotional expressions are differentiated early in the course of visual information processing, and that this remains true even when the stimuli are not consciously detected. Such findings corroborate the existence of a rapid, preattentive process, in which negative emotional stimuli initiate attentional capture more effectively than positive or neutral ones (Öhman et al., 2001; Vuilleumier, 2005; Maratos, 2011).

A number of studies have also addressed the temporal dynamics of attention shifting for emotional faces with some reports again claiming the presence of a very early effect. In a covert attention shifting paradigm using a bar-probe task with fearful, happy and neutral facial expressions as emotional cues (Pourtois et al., 2004), found an enhanced negative modulation of the C1 (80–100 ms) component for fearful compared to happy faces. Moreover, the P1 component was found to be enhanced for targets appearing in the former location of fearful faces confirming that fearful faces were efficient, and rapid attractors of attention. These results demonstrated that emotional features can modulate the neural activity in the striate cortex independently of spatial attention, prior to the N170. Nevertheless, previous (Clark et al., 1994) and subsequent (Rossi and Pourtois, 2013) studies have confirmed that the C1 is mainly generated in the striate and extrastriate visual cortices, and it can be sensitive to spatial (Vanlessen et al., 2012) and non-spatial (Proverbio et al., 2010) visual attention. For instance, selective attention on high and low spatial frequency gratings can increase respectively the negativity or the positivity of the C1, starting at 60 ms after the stimulus presentation (Zani and Proverbio, 2009), independently of the attended location. Conversely, the occipital P1 is modulated by spatial attention *per se* and in conjunction with non-spatial features (Zani and Proverbio, 2012). Moreover, the C1 component is sensitive to the valence of affective meaning of threatening compared to neutral stimuli (Stolarova et al., 2006). Nevertheless, the exact effect of emotional processing on the C1 component under subliminal conditions is still unclear.

An opposing view subsequently emerged to the one postulating a rapid, preattentive processing of emotional faces. This claimed that neural processing of emotional face stimuli requires some degree of attention for detection and processing to occur (Pessoa et al., 2002a,b, 2005a; Wronka and Walentowska, 2011). By manipulating the attentional load of concurrent tasks while presenting emotional faces (Erthal et al., 2005; Pessoa et al., 2005b; Yates et al., 2010), evidence was found showing that emotional stimuli were processed when the competing tasks required little attentional resources, but not when the attentional demand was high.

Recent studies have claimed that these two different views are not mutually exclusive, in view of the fact that attention and emotion interact to different degrees depending on perceptual and cognitive load, as well as on task demand (Okon-Singer et al., 2007). From this perspective, emotionally relevant stimuli would be processed automatically, in the sense that they do not require conscious monitoring, as long as sufficient attentional resources are available for processing to occur. Additionally, in a series of behavioral priming experiments (Finkbeiner and Palermo, 2009), it was found that non-emotional masked faces could be processed unconsciously even when spatial attention was not oriented toward them, contrary to non-face stimuli. These findings were further replicated by an another study confirming that, in comparison to non-faces, faces produced a priming effect regardless of spatial and temporal attention (Quek and Finkbeiner, 2013). These authors concluded that faces in general can be processed without awareness or attention.

In independent lines of study, separate investigations have addressed the electrophysiological correlates of visual awareness. Classically, several authors have put forward that the P300 may be linked to visual awareness (or more specifically P3b) since it is more pronounced when the stimulus are consciously perceived (Babiloni et al., 2006; Del Cul et al., 2007; Lamy et al., 2008). Conversely, other reports suggest that the P3b can rather reflect consequences of conscious perception (Rutiku et al., 2015), distinguishing between “perceptual awareness,” associated more to the attentional process of the visual stimuli, and “contextual awareness,” associated more to the working memory and context of face stimuli (Navajas et al., 2014). Others investigations have pointed to the possibility of an earlier component, arising closer to 230 ms. Indeed, in a series of studies, it was proposed that the P2 and the N2 may reflect the earliest activity linked to awareness and was consequently named “visual awareness negativity” or VAN (Koivisto and Revonsuo, 2008). This early posterior negative deflection, peaking around 200–250 ms after stimulus onset over lateral-occipital cortex (Koivisto and Revonsuo, 2003), is only elicited by stimuli that are presented above the subjective threshold of perception. This component has been found in different experimental paradigms using reduced visibility, such as masking, reduced-contrast stimuli, attentional blink, and change blindness (Koivisto and Revonsuo, 2010), and has been noted in conjunction with non-spatial (Koivisto and Revonsuo, 2008), as well as spatial attention (Koivisto et al., 2009). Evidence supports the hypothesis that the early part of VAN (130–200 ms) is modulated completely independently of attentional manipulation, whereas the later part (200–300 ms) is influenced by selective attention on posterior temporal sites, suggesting that the selection negativity (SN) and the VAN are dissociable (Koivisto and Revonsuo, 2007, 2008), with the later part of the VAN likely reflecting recurrent processing in the ventral visual stream (Vanni et al., 1996). However, the VAN and the P3 could represent simply different stages of conscious process, with the earliest phase reflecting the initial sensory aspect of conscious perception, and the later stage denoting the experience of the stimuli.

Despite the existing studies, the interactions between attention and awareness, and even more so with emotion, are still unclear due to the scarcity of studies specifically addressing this issue. In order to explore the interplay between awareness, attention, and emotion processing and to characterize their dynamics, we carried out a study in which we systematically varied the top-down contribution (defined as voluntary selective attention) and the bottom-up stimulus-driven contribution (defined as the extent of masking) in emotional face processing. Combining a selective attentional task (e.g., Proverbio et al., 2010) with a backward masking paradigm of different emotional categories, our intention was to determine: (i) how much processing could occur with limited visibility and without voluntary attention; and (ii) which type of differences arose across emotional expressions. Consequently, we recorded the EEG and computed ERPs during the central presentation of backward-masked face stimuli, depicting “negative” (i.e., fearful or angry) or “non-negative” (i.e., happy or neutral) expressions that were either attended or not (relevant or irrelevant to the task). Selective attention was manipulated by instructing participants to select a specific target category (e.g., “negative” stimuli) while ignoring the other category.

Four ERP components were examined: (i) the C1 considered the earliest index of emotional modulation (Pourtois et al., 2004) and object-based attention (Proverbio et al., 2010); (ii) the N170, considered to be an index of conscious (Batty and Taylor, 2003) and unconscious (Pegna et al., 2008) face processing; (iii) the P2, the N2 (VAN) and the P3, known to be as electrophysiological correlates of conscious access to visual stimuli (Railo et al., 2015); and (iv) the N2 and N3 which are possible indices of SN in conjunction with awareness (Koivisto et al., 2009).

## Materials and Methods

### Participants

Sixteen healthy volunteers took part in the EEG experiment (age range 19–33, mean = 24.25, SD = 4.33). All participants were right-handed as measured on the Oldfield–Edinburgh scale (Oldfield, 1971; mean laterality index: 13.9, range: 8–20) with normal or corrected-to-normal vision and gave their informed written consent prior to the procedure. The investigation was approved by the local Ethics Committee. Participants were paid 30 CHF for their contribution.

The group consisted of six women (age range 19–33, mean = 23.7) and 10 men (age range 19–33, mean = 24.8), mainly students of the University of Geneva and staff from Geneva University Hospital. Since anxiety is thought to influence behavioral and ERPs responses, especially with emotional stimuli (e.g., Holmes et al., 2009; Mühlberger et al., 2009; Putman, 2011), we administered the State/Trait Anxiety Inventory Test (STAI; Self-Evaluation Questionnaire of Spielberger et al., 1970) to all participants before every EEG recording session. This test measures anxiety levels in adults, differentiating between the temporary condition of “state anxiety” (S-A) and the more general and long-standing quality of “trait anxiety” (T-A). None of the participants presented a pathological level of anxiety (standard score mean: male group S-A ≈ 48, T-A ≈ 41; female group: S-A ≈ 42, T-A ≈ 44). During the ERP analysis, two participants were excluded from the experimental sample due to excessive artifacts.

### Apparatus and Stimuli

Black and white pictures of actors, displaying happy (H), angry (A), fearful (F), and neutral (Ne) facial expressions, were selected from a database that was previously set up^1^. The stimuli were modified by means of Adobe Photoshop 11, in order to remove hair, ears, and unwanted facial signs and to keep constant luminance values across emotional categories. Stimuli consisted of bitmap images of 6 cm × 6 cm (237 × 237-pixels) subtending a visual angle of 3° when viewed at a distance of 114 cm from the screen. We used 40 stimuli (20 adult faces representing males and 20 representing females) for each emotional condition (angry, fearful, happy, and neutral). We created cropped faces on a black equal background for every single emotional category. Scrambled faces, obtained by randomly scrambling 20 × 20-pixels squares on every single cropped face, were used as masks in the backward masking paradigm, thus preserving the same physicals parameters (Di Lollo et al., 2000). The total number of stimuli was 160 and each stimulus was presented 10 times for a total of 1600 trials. The run was composed of 10 blocks of 160 stimuli each and were displayed using E-prime^™^ software; the presentation of stimuli in every block, as well as the sequence of blocks and the response hand, were counterbalanced across participants and randomized within participants by the software.

### Design and Procedure

**FIGURE 1:**
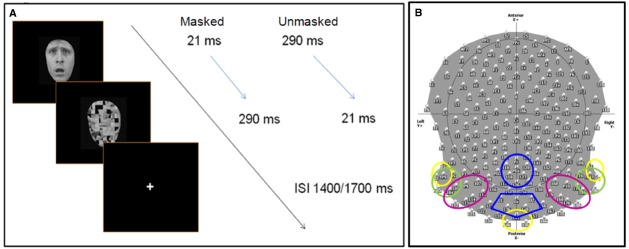
**(A)** Experimental procedure: face stimuli are presented for 21 ms (masked condition) or 290 ms (unmasked condition), followed immediately by the mask (scrambled face). The total duration (stimulus + mask) lasts 321 ms, with a inter stimulus interval between 1400 and 1700 ms, during which participants are allowed to give a manual response (key press). **(B)** The scalp distribution and names of 204 electrodes used during the EEG experiment. The colored circles delimit the different ROIs (region of interests) used to computed the ERP analysis. Each color refers to specific ROI(s) for specific components: C1 (40–100 ms)—yellow; N170 (140–190), N2 (subliminal 280–330 ms, supraliminal 240–320), N3 (320–390 ms)—green; P1 (95–135 ms), P2 (200–260 ms)—pink; P3 (380–580 ms)—blue. Electrodes within discrete ROIs are merged together.

Participants were comfortably seated in a moderately dark room (Faraday cage) while pictures were presented at the center of the screen. In order to manipulate voluntary selective attention, we used an attention task in which participants had to respond to a pre-defined category of stimuli by pressing a button on a keyboard, while ignoring the other categories. On half of the blocks, participants were asked to respond to happy and neutral stimuli (defined as “pleasant” faces), while on the other half, participants responded to fearful and angry stimuli (defined as “negative” faces; 50% in each category). Participants were instructed to respond as accurately and quickly as possible. During the EEG recording session, they were also asked to avoid any movement and to limit eye blinks. Before starting each sequence, the task instructions were indicated on the computer screen, and the participants were informed by the experimenter of the target category to which they had to reply. Target category varied randomly across blocks. Further, the experimenter verbally reiterated the instructions. The stimuli were presented for either 21 ms (“subliminal” presentation or masked condition) or for 290 ms (“supraliminal” presentation or unmasked condition), and were followed immediately by a mask constituted by a scrambled face. The duration of the mask was set such that the total stimulus duration (target + mask) was of 311 ms. Masks thus lasted respectively 290 and 21 ms (Figure 1A). In each sequence, half of the stimuli were presented subliminally. In the subliminal condition, it was emphasized that targets would be difficult to detect, but the participants were requested to focus on the stimulus, and to respond as soon as possible if they thought the target corresponded to the specified target category (pleasant or negative emotional expressions depending on the block).

Prior to the recording session, a training procedure was performed to familiarize the participants with the task and with the category of stimuli. Stimuli used in the training session were different to those used in the real experiment. However, paper printouts of all faces were presented to the participants once, before the EEG experiment to ensure that there was no ambiguity about the emotional expressions of the faces.

### Behavioral Data and Statistical Analysis

A repeated measure analysis of variance (two-way ANOVAs) within participants was applied on *d-prime values*, on *criterion rates* (c), and on *reaction times* (RTs), the latter only for target stimuli. We considered the following within factors: (i) *Presentation*: Masked and Unmasked; (ii) *Emotion*: Pleasant (Pls), Negative (Neg).

D-prime was used to evaluate the accuracy of the participants’ performance (signal detection theory, Macmillan and Creelman, 1991). This sensitivity rate was computed using hit and false alarm scores of every single subject in each category. Criterion rates were used to evaluate the willingness of the participant to make a false alarm. Defining the Criterion as the *z*-score on the Signal Absent Distribution, a high value of the Criterion implies that the respondent requires strong evidence before declaring that the signal is present.

### ERP Recordings and Analysis

Continuous EEG data were acquired at 1000 Hz using a Geodesics system (Electrical Geodesics, Inc., USA) with 256 equally-spaced scalp electrodes referenced to the vertex. Impendency was kept below 50 kΩ. ERPs were computed by Cartool software (http://sites.google.com/site/fbmlab/cartool, 3.40 versions). The EEG signal was filtered offline from 0.01 to 50 Hz. EEG was epoched offline from 100 ms before to 1400 ms after the onset of the stimulus face. Separate epochs were computed for each of the 16 stimulus categories using only correct responses, and were baseline corrected using a pre-stimulus interval of 100 ms prior to the onset of the stimulus. All the epochs contaminated by blinks, eye movements, or other artifacts (EEG sweeps with amplitudes exceeding ±100 μV) were excluded during the averaging procedure. Remaining artifacts were manually rejected upon visual inspection. During the ERP analysis, we systematically excluded 52 electrodes, situated over the face and in the most inferior part of the cap, decreasing their total number from 256 to 204 (Figure 1B). ERPs were then recalculated against the average reference (Lehmann and Skrandies, 1980).

We computed different region of interests (ROIs) based on different groups of electrodes, which were merged together (Figure 1B). We measured the peak amplitude and the latency of the following components:

• C1 (40–100 ms) over left temporal-occipital (83, 93, and TP9), right temporal-occipital (TP10, 191, and 201), and middle occipital (146, 147, and 156) regions (Figure 1B).
• N170 (140–190 ms), N2 (masked: 280–330 ms, unmasked: 240–320), and N3 (320–390 ms) over left occipital (93, TP9, 95, 103, 104, 105) and right occipital (177, 178, 189, TP10, 200, 201) regions.
• P1 (95–135 ms) and P2 (200–260 ms) over left occipital (104, 105, P9, 107) and right occipital (159, 160, 168, P10, 176, 177, 188, 189) regions.
• P3 (380–580 ms) over central parietal (110, 118, 126, 127, 128, POz, Pz) and occipital (136, 147, 148, O1, O2, Oz) scalp sites.

ERP amplitude and latency values were analyzed separately for each component, by means of four-way repeated-measures analyses of variance. We considered the following factors: (i) *Emotion (E)*: Angry (A), Happy (H), Fearful (F), Neutral (N); (ii) *Presentation*: Masked and Unmasked; (iii) Target category: Target (T), Non-target (NT); (iv) *ROIs*: left temporal-occipital (Lf), middle-occipital (Cx), right temporal-occipital (Rh) for the C1; left and right hemispheres for the P1, N170, P2, N2, and N3; we considered middle-parietal and middle-occipital areas for the P3. Additionally, we also computed separate repeated-measures ANOVAs (Emotion × Target Category × ROIs) for supraliminal and subliminal conditions to detect specific effects that could not emerge from the main ANOVA.

In behavioral and ERP statistical analyses, LSD tests were carried out for multiple mean *post hoc* comparisons in multiple ANOVA interactions. Greenhouse–Geisser corrections were applied to reduce the positive bias from repeated factors with more than two levels. We reported measures of effect size (${\text{η}}_{\text{p}}^{\text{2}}$) in addition to probability values.

## Results

### Behavioral Results

Participants’ performance, when discriminating facial expressions in the masked condition, was 46.7% (*z*-score value: 0.12), which is not significantly different from chance level (binomial distribution: *p* < 0.72). In the unmasked condition accuracy was at 85.4% (*z*-score value: –1.24).

The *d-prime* analysis showed that *d′* differed significantly between masked (0.78) and unmasked (2.16) stimuli [P factor: *F*_(1,13)_ = 98.84, MSE = 0.27, ε = 1, *p* < 0.0001, ${\text{η}}_{\text{p}}^{\text{2}}$ = 0.88] as it did for the *criterion* (*c*) [P factor: *F*_(1,13)_ = 9.99, MSE = 0.53, ε = 1, *p* < 0.008, ${\text{η}}_{\text{p}}^{\text{2}}$ = 0.50; subliminal: 0.66; supraliminal: 0.05].

Reaction times were significantly faster in response to unmasked than masked targets [*F*_(1,13)_ = 9.35, MSE = 11660, ε = 1, *p* < 0.009, ${\text{η}}_{\text{p}}^{\text{2}}$ = 0.41; mean values: Sup = 613 ms vs. Sub = 675 ms] and almost significantly faster to negative valence of faces [*F*_(1,13)_ = 4.11, MSE = 7825, ε = 1, *p* = 0.06, ${\text{η}}_{\text{p}}^{\text{2}}$ = 0.24; mean values: Pls = 661 ms and Neg = 627 ms]. More specifically, the slight effect between positive and negative valence was exclusively due to the neutral faces as shown by the significant effects in the repeated-measures ANOVAs with four separate emotions [*F*_(3,39)_ = 10.79, MSE = 11637, ε = 0.78, *p* < 0.0002, ${\text{η}}_{\text{p}}^{\text{2}}$ = 0.45; mean values: *A* = 626 ms, *F* = 629 ms, and *H* = 608 ms vs. *N* = 714 ms, *post hoc* comparisons: *p*s < 0.0002].

### Electrophysiological Results

#### C1 Component

***Latency***

An earlier peak was detected for: (i) non-targets (63 ms) compared with targets (68 ms) in the masked presentation (*p* < 0.01); masked (63 ms) compared to unmasked (67 ms) non-target stimuli (*p* < 0.02); unmasked (64 ms) compared to masked (68 ms) target stimuli (*p* < 0.05), as shown by the interaction of “Presentation × Target Category” [*F*_(1,13)_ = 12, MSE = 194, ε = 1, *p* < 0.004, η^2^ = 0.48]. Masked stimuli presented an earlier peak over right (64 ms) than left (67 ms) electrodes, whereas unmasked stimuli showed a later peak over central (67 ms) than left (64 ms) electrodes [“Presentation × ROI”: *F*_(2,26)_ = 3.66, MSE = 103, ε = 0.85, *p* < 0.05, ${\text{η}}_{\text{p}}^{\text{2}}$ = 0.22].

*Masked presentation*. The same attentional effect, found in the main ANOVAs, occurred in this analysis [“Target Category”: *F*_(1,13)_ = 6.1, MSE = 1.28, ε = 1, *p* < 0.03, ${\text{η}}_{\text{p}}^{\text{2}}$ = 0.32] showing an earlier peak for non-targets (63 ms) than targets (68 ms) stimuli.

*Unmasked presentation*. No significant result was found.

***Peak***

The interaction of “Emotion × Target Category” was significant [*F*_(3,39)_ = 2.8, MSE = 1.28, ε = 1, *p* = 0.05, ${\text{η}}_{\text{p}}^{\text{2}}$ = 0.17], showing a difference between targets (–0.98 μV) and non-targets (–0.4 μV) only in the fearful condition (*post hoc* comparisons: *p*s < 0.003), and between fearful and happy (–0.54 μV), as well as angry and neutral (both –0.55 μV) faces in the attentive condition (*post hoc* comparisons *p*s < 0.002).

*Masked presentation*. The amplitude was affected by the interaction of “Emotion × Target Category” [*F*_(3,39)_ = 3, MSE = 3.06, ε = 1, *p* < 0.05, ${\text{η}}_{\text{p}}^{\text{2}}$ = 0.19], showing an increase of negativity for fearful (–1.49 μV) compared to angry (–0.57 μV), happy (–0.44 μV; *p* < 0.02), and neutral faces (–0.45 μV; *p*s < 0.01) only in the attentive condition. Moreover, only fearful faces elicited a greater negativity between targets (–1.49 μV) and non-targets (–0.35 μV; *p* < 0.005).

*Unmasked presentation*. No significant result was found.

#### P1 Component

***Latency***

Negative facial expressions elicited an earlier peak compared with pleasant emotional faces [“Emotion”: *F*_(3,39)_ = 13.33, MSE = 66, ε = 0.65, *p* < 0.0002, ${\text{η}}_{\text{p}}^{\text{2}}$ = 0.5; mean values: *A* = 116 ms and *F* = 114 ms vs. *H* = 120 ms and *N* = 119 ms; *post hoc* comparisons: *p*s < 0.009].

*Masked presentation*. Fearful faces elicited an earlier peak compared with all the other emotional expressions [“Emotion”: *F*_(3,39)_ = 11.12, MSE = 70, ε = 0.62, *p* < 0.0005, ${\text{η}}_{\text{p}}^{\text{2}}$ = 0.46; mean values: *A* = 118 ms, *F* = 113 ms, *H* = 122 ms, *N* = 120 ms; *post hoc* comparisons: *p*s < 0.01]. Moreover, the difference between angry and happy faces, as well as between angry and neutral faces, was significant (*post hoc* comparisons: *p*s < 0.0001).

*Unmasked presentation*. Negative facial expressions elicited an earlier peak compared with pleasant emotional faces [“Emotion”: *F*_(3,39)_ = 4.77, MSE = 50, ε = 0.86, *p* < 0.009, ${\text{η}}_{\text{p}}^{\text{2}}$ = 0.27; mean values: *A* = 115 ms and *F* = 115 ms vs. *H* = 118 ms and *N* = 119 ms; *post hoc* comparisons: *p*s < 0.04].

***Peak***

No significant result was found in the main ANOVA, or in the ANOVA of single presentation.

#### N170 Component

***Latency***

Masked stimuli elicited an earlier peak compared to unmasked faces [“Presentation”: *F*_(1,13)_ = 12.1, MSE = 118, ε = 1, *p* < 0.004, ${\text{η}}_{\text{p}}^{\text{2}}$ = 0.48; mean values: 163 vs. 167 ms].

*Masked presentation*. Negative (anger and fear) facial expressions elicited an earlier peak compared to pleasant (happiness and neutral) faces [“Emotion”: *F*_(3,39)_ = 4.84, MSE = 116, ε = 0.51, *p* < 0.03, ${\text{η}}_{\text{p}}^{\text{2}}$ = 0.27; *A* = 162 ms and *F* = 161 ms vs. *H* ≈ *N* = 167 ms; *post hoc* comparisons: *p*s < 0.05].

*Unmasked presentation*. “Emotion” factor [“Emotion”: *F*_(3,39)_ = 6.74, MSE = 47, ε = 0.84, *p* < 0.001, ${\text{η}}_{\text{p}}^{\text{2}}$ = 0.34] showed a later peak for happy (169 ms) than fear (165 ms) and angry (163 ms) facial expressions, and for neutral (167 ms) compared to angry faces (*post hoc* comparisons: *p*s < 0.01). Moreover, this component peaked earlier on the right (164 ms) than left (168 ms) hemisphere [“ROI”: *F*_(1,13)_ = 4.9, MSE = 216, ε = 1, *p* < 0.045, η^2^ = 0.27].

***Peak***

**FIGURE 2:**
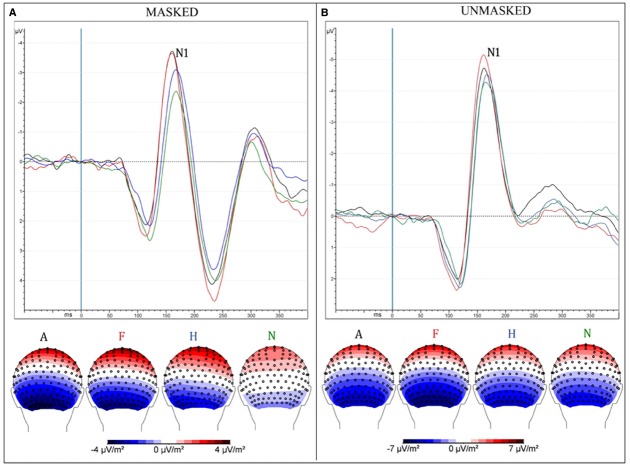
**(A)** Masked presentation; **(B)** Unmasked presentation. Both figures depict grand average ERPs, merged across electrodes ROI of N170 and between attentive and inattentive conditions (above); Scalp-Current-Density maps between 160 and 190 ms after the presentation of emotional stimuli, corresponding to N170 time window (below). Each ERP (and map) represents a different emotional condition: Anger (A—black), Fear (F—red), Happiness (H—blue), neutral (N—green).

Unmasked pictures produced a greater amplitude compared to masked faces [“Presentation”: *F*_(1,13)_ = 31.35, MSE = 9.75, ε = 1, *p* < 0.0001, ${\text{η}}_{\text{p}}^{\text{2}}$ = 0.71; mean values: –5.7 and –4 μV]. Negative facial expressions increased significantly the amplitude of this component compared to pleasant faces [“Emotion”: *F*_(3,39)_ = 6.38, MSE = 4.99, ε = 0.58, *p* < 0.009, ${\text{η}}_{\text{p}}^{\text{2}}$ = 0.33; mean values: *A* = –5.24 μV and *F* = 5.36 μV vs. *H* = –4.63 μV and *n* = –4.23 μV; *post hoc* comparisons: *p*s < 0.05]. The interaction of “Presentation × Emotions” [*F*_(3,39)_ = 4.1, MSE = 2.56, ε = 0.73, *p* < 0.025, ${\text{η}}_{\text{p}}^{\text{2}}$ = 0.24] progressively showed an increased negativity across emotions, from neutral to angry faces (*N* = –3 μV < *H* = –3.79 μV < *F* = –4.6 μV and *A* = –4.77 μV; *p*s < 0.02) only in the masked condition (Figure 2). In the unmasked condition, only fearful faces (–6.12 μV) elicited a greater negativity compared to happy (–5.48 μV) and neutral facial (–5.44 μV) expressions (*post hoc* comparisons: *p*s < 0.04).

*Masked presentation*. Angry and fearful facial expressions elicited a greater negativity compared with happy and neutral faces [“Emotion”: *F*_(3,39)_ = 9.26, MSE = 4, ε = 1, *p* < 0.0001, ${\text{η}}_{\text{p}}^{\text{2}}$ = 0.42; *A* = –4.77 and *F* = –4.6 < *H* = –3.79 < *N* = –3; *post hoc* comparisons: *p*s < 0.05].

*Unmasked presentation*. No significant result was found.

#### P2 Component

***Latency***

Targets elicited an earlier peak compared to non-targets [“Target Category”: *F*_(1,13)_ = 6.6, MSE = 236, ε = 0.1, *p* < 0.024, ${\text{η}}_{\text{p}}^{\text{2}}$ = 0.34; mean values: 231 vs. 234 ms respectively], as well unmasked in comparison to masked stimuli [“Presentation”: *F*_(1,13)_ = 5.89, MSE = 242, ε = 1, *p* < 0.03, η^2^ = 0.32; mean values: 231 vs. 235 ms respectively]. This component peaked earlier over right than left leads, as proved by “ROI” factor [*F*_(1,13)_ = 6.89, MSE = 134, ε = 1, *p* < 0.02, ${\text{η}}_{\text{p}}^{\text{2}}$ = 0.35; mean values: 231 vs. 234 ms respectively].

*Masked presentation*. “Target Category” factor was significant [*F*_(1,13)_ = 6.64, MSE = 220, ε = 1, *p* < 0.023, ${\text{η}}_{\text{p}}^{\text{2}}$ = 0.34; mean values: T = 232 ms vs. NT = 237 ms], as well as “ROI” *per se* [*F*_(1,13)_ = 9.53, MSE = 54, ε = 1, *p* < 0.009, η^2^ = 0.42; mean values: Rh = 233 ms vs. Lf = 236 ms].

*Unmasked presentation*. No significant result was found.

***Peak***

**FIGURE 3:**
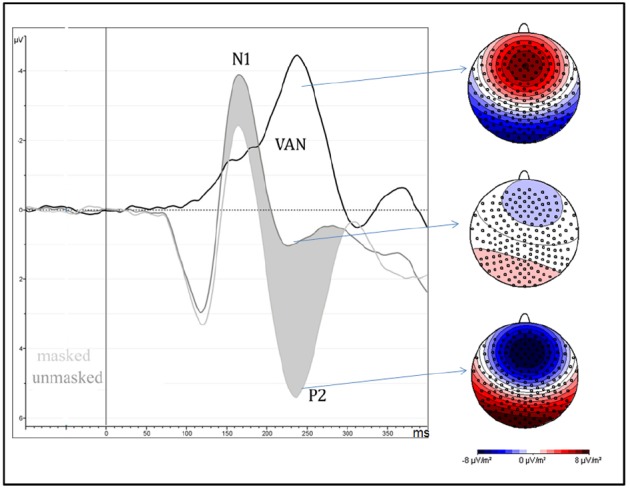
**It depicts the strong negative effect (VAN = visual awareness negativity) between 200 and 260 ms after the stimulus onset, over occipital brain regions, and are computed as the difference wave between masked and unmasked stimuli, independently of attentional condition and type of emotion.** On the right side of the figure, scalp-current-density maps, computed in the same time window, show the electrical scalp distribution of masked (bottom), unmasked (middle) stimuli and their difference (top).

Amplitude was greater for unmasked (6.1 μV) than masked (1.6 μV) stimuli as shown by means of “Presentation” factor *per se* [*F*_(1,13)_ = 51.85, MSE = 41, ε = 1, *p* < 0.0001, η^2^ = 0.8; see Figure 3].

*Masked presentation*. No significant result was found.

*Unmasked presentation*. No significant result was found.

#### N2 Component

***Latency***

The peak was earlier for unmasked (281 ms) than masked (304 ms) [“Presentation”: *F*_(1,13)_ = 51.45, MSE = 1147, ε = 1, *p* < 0.0001, η^2^ = 0.80]. The interaction with “Emotion” factor showed that each emotional category of unmasked faces elicited a earlier peak compared to the same emotional category of masked stimuli [*F*_(3,39)_ = 3.83, MSE = 151, ε = 0.73, *p* < 0.03, ${\text{η}}_{\text{p}}^{\text{2}}$ = 0.23; mean values: A—276 ms vs. 305 ms; F—283 ms vs. 304 ms; H—279 ms vs. 304 ms; N—285 ms vs. 303 ms; *p*s < 0.0001].

*Masked and unmasked presentation*. No significant result was found.

***Peak***

Amplitude was significantly different in the Target Category factor only in the masked presentation, where targets (–1.89 μV) elicited a greater amplitude than non-targets (–0.97 μV), as shown by the *post hoc* comparisons (*p* < 0.002) of the “Presentation × Target Category” interaction [*F*_(1,13)_ = 5.65, MSE = 3.14, ε = 1, *p* < 0.04, ${\text{η}}_{\text{p}}^{\text{2}}$ = 0.3].

*Masked presentation*. The “Target Category” factor [*F*_(1,13)_ = 7.42, MSE = 6.34, ε = 1, *p* < 0.02, ${\text{η}}_{\text{p}}^{\text{2}}$ = 0.36] indicated that target (–1.89) stimuli increased the negative amplitude more than the non-target (–0.97) ones.

*Unmasked presentation*. No significant result was found.

#### N3 Component

***Latency***

No significant result was found in the separate ANOVAs computed for each type of presentation.

***Peak***

Masked stimuli elicited a smaller negativity compared with unmasked faces [“Presentation”: *F*_(1,13)_ = 32.7, MSE = 20, ε = 1, *p* < 0.0001, ${\text{η}}_{\text{p}}^{\text{2}}$ = 0.72; mean values: 1.87 vs. –0.53 μV], as well as non-targets compared to targets, independently of the type of presentation [“Target Category”: *F*_(1,13)_ = 12.12, MSE = 2.88, ε = 1, *p* < 0.004, ${\text{η}}_{\text{p}}^{\text{2}}$ = 0.48; mean values: 1 vs. 0.39 μV respectively]. This component was greater on the left than right hemisphere [“ROI”: *F*_(1,13)_ = 6.28, MSE = 9.74, ε = 1, *p* < 0.03, ${\text{η}}_{\text{p}}^{\text{2}}$ = 0.33; mean values: 0.3 vs. 1 μV respectively].

*Masked presentation*. The factor “Target Category” was significant, revealing a greater negativity for targets compared to non-targets [*F*_(1,13)_ = 14.2, MSE = 1.12, ε = 1, *p* < 0.003, ${\text{η}}_{\text{p}}^{\text{2}}$ = 0.52; mean values: *T* = 1.6 μV vs. NT = 2.1 μV].

*Unmasked presentation*. The factor “Target Category” was again significant, revealing a greater negativity for targets compared with non-targets [*F*_(1,13)_ = 5.67, MSE = 3.36, ε = 1, *p* < 0.04, ${\text{η}}_{\text{p}}^{\text{2}}$ = 0.3; mean values: *T* = –0.83 μV vs. NT = –0.24 μV].

#### P3 Component

***Latency***

“Target Category” factor was significant *per se*, showing an earlier peak for non-target than for target stimuli [*F*_(1,13)_ = 8.54, MSE = 1001, ε = 1, *p* < 0.02, ${\text{η}}_{\text{p}}^{\text{2}}$ = 0.40; mean values: 473 vs. 482 ms respectively]. This component peaked earlier over occipital than parietal leads [“ROI” factor: *F*_(1,13)_ = 5.77, MSE = 5210, ε = 1, *p* < 0.032, ${\text{η}}_{\text{p}}^{\text{2}}$ = 0.31; mean values: 469 vs. 485 ms respectively].

*Masked presentation*. No significant result was found.

*Unmasked presentation*. “Target Category” factor was significant *per se*, showing an earlier peak for non-target than for target stimuli [*F*_(1,13)_ = 7, MSE = 1205, ε = 1, *p* < 0.02, ${\text{η}}_{\text{p}}^{\text{2}}$ = 0.35; mean values: 472 vs. 485 ms respectively].

***Peak***

**FIGURE 4:**
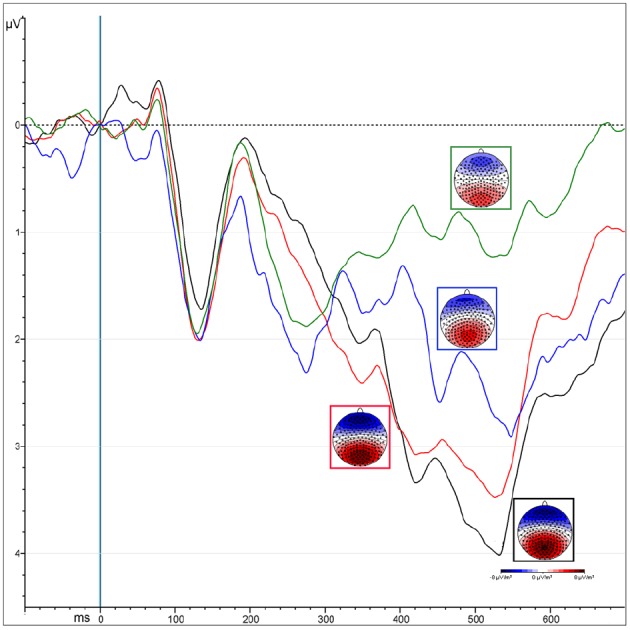
**ERPs representing the P300 amplitude over central parietal leads, computed by averaging the electrodes of its specific ROI.** Colors correspond to different conditions coming from the interaction of “attention” and “presentation” factors: unmasked targets (black) and non-targets (red); masked targets (blue) and non-targets (green).

The “Presentation” factor was significant [*F*_(1,13)_ = 33.53, MSE = 12.85, ε = 1, *p* < 0.0001, ${\text{η}}_{\text{p}}^{\text{2}}$ = 0.72], showing increased amplitude for unmasked (5.2 μV) than masked (3.24 μV) emotional expressions (Figure 4). Targets elicited greater positivity compared to non-targets [“Attention”: *F*_(1,13)_ = 12.5, MSE = 3.38, ε = 1, *p* < 0.004, ${\text{η}}_{\text{p}}^{\text{2}}$ = 0.5]. This effect was observed only over parietal areas (4.47 vs. 3.49 μV, *p* < 0.0002), but not over occipital areas (4.3 vs. 4.6 μV, *p* = 0.2), as shown by the interaction of “Attention × ROI” [*F*_(1,13)_ = 7.66, MSE = 1.9, ε = 1, *p* < 0.02, ${\text{η}}_{\text{p}}^{\text{2}}$ = 0.37].

*Masked presentation*. The amplitude was significantly more positive for target than non-target stimuli [“Attention”: *F*_(1,13)_ = 7.4, MSE = 6.67, ε = 1, *p* < 0.02, ${\text{η}}_{\text{p}}^{\text{2}}$ = 0.5; mean values: 3.71 and 2.77 μV respectively].

*Unmasked presentation*. Amplitudes were increased for targets compared to non-targets only over parietal leads, as revealed by the interaction of “Attention × ROI” [*F*_(1,13)_ = 4.98, MSE = 0.94, ε = 1, *p* < 0.04, ${\text{η}}_{\text{p}}^{\text{2}}$ = 0.28; mean values occipital ROI: *T* = 5.2 μV vs. NT = 4.6 μV, *p* < 0.008; mean values occipital ROI: *T* = NT = 5.49 μV].

## Discussion

Our study reveals that facial expressions can be processed without awareness and independently of whether participants are engaged in an attempt to detect a specific emotion. Additionally, our results highlight that top-down selective attention and awareness of these stimuli operate in distinct time periods.

In both masked and unmasked conditions, the N170 showed an increased negative amplitude in response to negative faces compared to happy and neutral faces, revealing that the initial processing of emotion occurs independently of awareness (Figure 2). The P2 component was found to be linked to stimulus visibility, independently of attention and emotion, while the P3 and N3 showed an interaction between awareness and selective attention. Finally, the N2 and N3 were greater for targets than non-targets, independently of facial expression and type of presentation (masked vs. unmasked). At the N2 level, an enhanced response for targets emerged only in the masked condition, while, at the N3 level, the effect was observed for both masked and unmasked stimuli.

Our study therefore confirms previous findings, demonstrating that emotional expressions are processed without awareness. Furthermore, this process occurs even when the emotion is unattended. However, attention can modulate conscious processing at a very early stage (within 100 ms after the stimulus onset according to our findings), depending on the type of emotional stimulus. Interestingly, we found that attention and awareness for these emotional stimuli arise at different time periods and rely on different networks with respect to those involved in the initial stage.

### Temporal Processing of Emotions

Evidence from electrophysiological studies using implicit tasks, supported a model of automatic (defined here as occurring without voluntary attention), and rapid processing of emotional expressions (Batty and Taylor, 2003), suggesting that the N170 can be linked to the processing of emotional faces. In their study, Batty and Taylor (2003) investigated six basic emotional expressions (sadness, fear, disgust, anger, happiness, and surprise) as well as neutral faces, and found that the N170 was modulated by emotion and produced a significantly larger amplitude for fearful compared to other expressions. Blau et al. (2007) also observed an enhanced N170 amplitude for fearful compared to neutral faces under non-explicit task conditions. They showed that the processing of facial structure and emotion produces electrophysiological responses within the same time interval, suggesting that the emotion processing does not occur solely after a supposed initial encoding period, as previously reported (Eimer and Holmes, 2002; Ashley et al., 2004). Separate investigations concluded that emotional expressions modulate the N170, even in the absence of visual awareness. As noted above, a greater activation for negative emotions has been found for backward-masked faces presented below the threshold of visual awareness (Pegna et al., 2008). The same time-window for subliminal processing of emotions was identified between 140 and 180 ms by Kiss and Eimer (2008), who described an enhanced response for fear compared to happy faces in supraliminal and subliminal conditions over frontal and central sites. Thus, it seems clear that a strong modulatory activity occurs very rapidly in response to the emotional valence, even when the stimulus is not accessible to perceptual awareness.

Findings evidencing an N170 modulation for undetectable faces were replicated in a study using supraliminal and subliminal faces in which emotions were irrelevant to the task (Pegna et al., 2011). The results showed that the N170 was enhanced both for detectable and undetectable fearful faces, and that this occurred even when the participants were engaged in an orthogonal task (i.e., comparing lateral flanker bars). Consequently, the N170 modulation to emotional expressions is not eliminated when the stimuli are unattended. In a previous MEG study (Bayle and Taylor, 2010), demonstrated that the M170 was sensitive to emotions whether or not they were attended (although different areas appeared to be involved in each case). This contradicts certain previous findings such as those of Wronka and Walentowska (2011) who investigated emotional face processing using a selective attentive task and reported an effect on the N170, but only when the participants were asked to respond to the emotional expression of the face. Indeed, when requested to judge the gender of the faces the effect disappeared. These authors argued that top-down attentional control mechanisms were required for emotional processing in accordance with previous reports (Pessoa et al., 2002a,b). Although this conclusion has been the subject of much debate, our study corroborates reports suggesting that the N170 is modulated by emotions without attention or awareness, and prior to their engagement.

Interestingly, we also found a modulation at the C1 (N70) level for fearful faces, which showed an increased negativity compared to the other emotions. The early effect of emotion arising at 70 ms, may be due to the salience of fearful faces in conjunction with top-down mechanisms of voluntary attention that respond to visual features pertaining to the specific type of stimulus. This would explain why in our case, even under conditions of limited visibility, participants, focusing on threatening stimuli, produced an enhanced C1. However, the effect was found only in the masked attentive condition, suggesting that “fear” might capture attention solely during brief presentations of stimuli (i.e., 21 ms in our condition). In support of this explanation, similar effects have been described by Bannerman et al. (2010) who found a comparable outcome using a spatial cueing paradigm and measuring visual saccades and manual RTs to the cued locations. Fearful or neutral body expressions were used as cues which were presented for brief (20 ms) or long (100 ms) durations. These were followed by targets to which participants were asked to respond, either with a button press or with a saccade. In the short presentations, no differences were found in manual responses across emotional cues. However, longer presentations produced an emotion-dependant effect. Importantly, saccadic RTs were significantly faster for fearful compared to neutral emotional expressions in validly-cued locations, only during brief presentations. Thus, shorter presentations may enhance the effect of threatening cues, at least when saccadic responses are considered, a finding that could corroborate our results for the C1 response.

In behavioral paradigms, Quek and Finkbeiner (2013) confirm that masked non-emotional faces are processed even when they are not attended. Our ERP data additionally show this effect on the emotional valence, in agreement with other ERP (e.g., Pegna et al., 2011) and fMRI studies (e.g., Vuilleumier et al., 2001). Interestingly, our findings also reveal that selective attention has a differential effect on the extent of unconscious processing depending on the different emotions. Indeed, selective attention can boost the processing of threatening faces in the masked condition, suggesting that unconscious processes can modulate selective attention in return. This view adopts a middle position between the complete independence of awareness and attentional resources (e.g., Posner and Snyder, 1975; McCormick, 1997), and the influence of attention on the engagement of cognitive resources in unconscious processing (e.g., Dehaene et al., 2006). However, the intentions of participants and their action plans can influence the initial unconscious processing of visual stimuli, as proposed by Neumann (1984). This would suggest that the early process is likely to be modulated by top-down strategic control and the unconscious processing of visual stimuli can be defined as “automatic” inasmuch as it does not hand out information necessary to support strategic processing steps (for a complete review, see Kiefer, 2007). Additionally, it has been demonstrated that the specific instructions given to participants prior to performing a task (Van den Bussche et al., 2009), as well as the knowledge of a prime or stimulus before starting an experiment (Al-Janabi and Finkbeiner, 2012), can increase subsequent masked cueing effects, enhancing the perception and the discrimination of unseen stimulus and boosting invisible objects into consciousness (Lin and Murray, 2014). It may be argued that in our case, the exposure to the stimuli before the experimental session, as well as their random presentations below and above the threshold of visibility, could have enhanced the emotional effect in the subliminal condition. However, this top-down influence seems to appear solely with negative emotions, thus precluding this interpretation.

### Temporal Processing of Attention and Awareness

No effects were observed on the P1 component in response to emotion, selective attention or awareness. On the other hand, the P2 was affected by stimulus visibility but showed no modulation associated with attention (Figure 3), while the N2 showed an interaction of attention with the visibility of the stimuli, the response being modulated by selective attention only when the target was clearly visible. The N3 and the P3 however, produced greater responses for targets compared to non-targets in the masked as well as in the unmasked condition (Figure 4), revealing neural activity linked essentially to selective attention during this period. Thus, the neural mechanisms of visual attention and visual awareness seem to be independent and dissociated in the initial periods, with awareness arising slightly earlier in time.

Our results therefore corroborate previous suggestions that SN and awareness (the VAN) are dissociable (Koivisto and Revonsuo, 2007, 2008; Railo et al., 2015), as selective attention did not modulate the first period of our awareness-related component occurring between 200 and 260 ms after stimulus onset (the P2 component). On the other hand, attentional effects appeared at the N2 and at the N3 level, only 280 ms after the presentation of the stimulus. This view is by no means generally agreed upon. For instance, Shafto and Pitts (2015) showed that the N170, the VAN and the P300 were absent during inattentional blindness, but were present and were modulated in the aware condition, when faces were task-relevant. They claim that selective attention and perceptual awareness are distinct and separable processes, but only singularly dissociable, meaning that attention can operate even in the absence of awareness, while perceptual awareness cannot operate without attention. Their results argue against the hypothesis of the P3 wave as a neural marker of workspace activation and conscious access (Dehaene et al., 2014). Evidently, it is not always justified to assume that the VAN indexes visual perception alone, due to the fact that attentional ERP components present similar latencies and topographies as the VAN, leading to difficulties in distinguishing them from one another (Rutiku et al., 2015). In addition, the type of paradigm employed (e.g., feature-based vs. spatial attention, stimulus expectation, and adaptation or storing in working memory) may lead to differences in accessibility to consciousness by the visual stimuli. Thus, the prerequisites and consequences of consciousness may become confused with awareness *per se* and its actual neural markers (Aru et al., 2012; De Graaf et al., 2012).

In our case, the P2, corresponding here to the first part of the VAN (200–260 ms), was shown to emerge independently of top-down selection. Awareness and selective attention began to interact at the N2 level, likely corresponding to the second part of the VAN, showing the greatest interaction effect on the parietal P3. The N3 and the P3 waves showed an effect linked to the detectability of stimuli independently of the emotional valence and, thus, dependent on top-down mechanisms (voluntary attention). This suggests that the P300 should rather be seen as a consequence of consciousness, related for example to post-perceptual processing, rather than to awareness *per se* (Shafto and Pitts, 2015).

## Conclusion

In the literature, it has been shown that both emotional faces and awareness can affect the amplitude (Balconi and Lucchiari, 2007) and latency (Balconi and Mazza, 2009) of the N2, while threatening stimuli appear to enhance the N170 (Pegna et al., 2008, 2011). The increased amplitudes for such stimuli are interpreted as a heightened activity in response to their emotional content, while the delayed peak for the masked stimuli may reflect the effort necessary to compute a weaker stimulus. These findings are in line with the view that emotional stimuli are capable of capturing attention and eliciting a rapid, preattentive response in the absence of awareness (Öhman et al., 2000, 2001; Vuilleumier, 2005; Maratos, 2011) and is consistent with the hypothesis of dual pathways for visual processing, which includes a subcortical pathway through the superior colliculus and pulvinar to the amygdala allowing a rapid response to signals of threat is required (Liddell et al., 2004, 2005; LeDoux, 2007).

Finally, the pattern of effects, observed in the ERPs, appears to be in line with the “cumulative influence model” put forward by (Tallon-Baudry, 2012). This model states that attention and awareness might be initially independent and combined later on, when the response of the subject reaches the decisional stage. In our study, attention and awareness are indeed not initially combined, as the latter emerges after around 200 ms before any effect of selective attention is observed (i.e., targets and non-targets do not differ). On the other hand, effects of selective attention, appear after 280 ms, first in interaction with stimulus visibility, but then independently. The fact that the neural signatures of awareness and of selective attention are distinct, albeit partially, argues in favor of their relative independence and suggest that both can contribute to the final decisional processes. That said, selective attention appears to be influenced by the emotional nature of the stimuli, which in turn impinges on unconscious processing at a very early stage (Finkbeiner and Palermo, 2009).

### Conflict of Interest Statement

The authors declare that the research was conducted in the absence of any commercial or financial relationships that could be construed as a potential conflict of interest.

## Footnotes

1 Previously, 46 volunteers (24 females and 22 males) classified the type of emotion of 130 faces taken from different database by means of a taxonomical scale of six basic emotions as defined by Ekman and Friesen (1971): anger, happiness, fear, surprise, disgust, and sadness (plus neutral). In the EEG experiment, we selected only those pictures reaching the threshold of 70% of consensus.

## References

[B1] Al-Janabi S. Finkbeiner M. (2012). Effective processing of masked eye gaze requires volitional control. Exp. Brain Res. 216, 433–443. 10.1007/s00221-011-2944-0 22101495

[B2] Aru J. Bachmann T. Singer W. Melloni L. (2012). Distilling the neural correlates of consciousness. Neurosci. Biobehav. Rev. 36, 737–746. 10.1016/j.neubiorev.2011.12.003 22192881

[B3] Ashley V. Vuilleumier P. Swick D. (2004). Time course and specificity of event-related potentials to emotional expressions. Neuroimage 15, 211–216. 10.1097/00001756-200401190-00041 15106860

[B4] Babiloni C. Vecchio F. Miriello M. Romani G. L. Rossini P. M. (2006). Visuo-spatial consciousness and parieto-occipital areas: a high-resolution EEG study. Cereb. Cortex 16, 37–46. 10.1093/cercor/bhi082 15800023

[B5] Balconi M. Lucchiari C. (2007). Consciousness and emotional facial expression recognition: subliminal/supraliminal stimulation effect on n200 and p300 ERPs. J. Psychophysiol. 21, 100–108. 10.1027/0269-8803.21.2.100

[B6] Balconi M. Mazza G. (2009). Consciousness and emotion: ERP modulation and attentive vs. pre-attentive elaboration of emotional facial expressions by backward masking. Motiv. Emot. 33, 113–124. 10.1007/s11031-009-9122-8

[B7] Bannerman R. L. Milders M. Sahraie A. (2010). Attentional bias to brief threat-related faces revealed by saccadic eye movements. Emotion 10, 733–738. 10.1037/a0019354 21038958

[B8] Batty M. Taylor M. J. (2003). Early processing of the six basic facial emotional expressions. Cogn. Brain Res. 17, 613–620. 10.1016/S0926-6410(03)00174-5 14561449

[B9] Bayle D. J. Taylor M. J. (2010). Attention inhibition of early cortical activation to fearful faces. Brain Res. 1313, 113–123. 10.1016/j.brainres.2009.11.060 20004181

[B10] Blau V. Maurer U. Tottenham N. McCandliss B. (2007). The face-specific N170 component is modulated by emotional facial expression. Behav. Brain Funct. 3, 7. 10.1186/1744-9081-3-7 17244356 PMC1794418

[B11] Bocanegra B. R. Zeelenberg R. (2009). Emotion improves and impairs early vision. Psychol. Sci. 20, 707–713. 10.1111/j.1467-9280.2009.02354.x 19422624

[B12] Clark V. P. Fan S. Hillyard S. A. (1994). Identification of early visual evoked potential generators by retinotopic and topographic analyses. Hum. Brain Mapp. 2, 170–187. 10.1002/hbm.460020306

[B13] De Graaf T. A. Hsieh P.-J. Sack A. T. (2012). The ‘correlates’ in neural correlates of consciousness. Neurosci. Biobehav. Rev. 36, 191–197. 10.1016/j.neubiorev.2011.05.012 21651927

[B14] Dehaene S. Changeux J. P. Naccache L. Sackur J. Sergent C. (2006). Conscious, preconscious, and subliminal processing: a testable taxonomy. Trends Cogn. Sci. 10, 204–211. 10.1016/j.tics.2006.03.007 16603406

[B15] Dehaene S. Charles L. King J.-R. Marti S. (2014). Toward a computational theory of conscious processing. Curr. Opin. Neurobiol 25, 76–84. 10.1016/j.conb.2013.12.005 24709604 PMC5635963

[B16] Del Cul A. Baillet S. Dehaene S. (2007). Brain dynamics underlying the nonlinear threshold for access to consciousness. PLoS Biol. 5:e260. 10.1371/journal.pbio.0050260 17896866 PMC1988856

[B17] Del Zotto M. Deiber M. P. Legrand L. B. De Gelder B. Pegna A. J. (2013). Emotional expressions modulate low *α* and *β* oscillations in a cortically blind patient. Int. J. Psychophysiol. 90, 358–362. 10.1016/j.ijpsycho.2013.10.007 24144636

[B18] Di Lollo V. Enns J. T. Rensink R. A. (2000). Competition for consciousness among visual events: the psychophysics of reentrant visual processes. J. Exp. Psychol. Gen. 129, 481–507. 10.1037/0096-3445.129.4.481 11142864

[B19] Eimer M. Holmes A. (2002). An ERP study on the time course of emotional face processing. Neuroimage 13, 427–431. 10.1097/00001756-200203250-00013 11930154

[B20] Ekman, P. and Friesen W. V. (1971). Constants across cultures in the face and emotion. J. Personal. Soci. Psychol. 17, 124–129. 5542557 10.1037/h0030377

[B21] Erthal F. T. De Oliveira L. Mocaiber I. Pereira M. Machado-Pinheiro W. Volchan E. (2005). Load-dependent modulation of affective picture processing. Cogn. Affect. Behav. Neurosci. 5, 388–395. 10.3758/CABN.5.4.388 16541809

[B22] Finkbeiner M. Palermo R. (2009). The role of spatial attention in nonconscious processing: a comparison of face and nonface stimuli. Psychol. Sci. 20, 42–51. 10.1111/j.1467-9280.2008.02256.x 19152540

[B23] Gonzalez Andino S. L. Grave de Peralta Menendez R. Khateb A. Landis T. Pegna A. J. (2009). Electrophysiological correlates of affective blindsight. Neuroimage 44, 581–589. 10.1016/j.neuroimage.2008.09.002 18835454

[B24] Holmes A. Nielsen M. Tipper S. Green S. (2009). An electrophysiological investigation into the automaticity of emotional face processing in high versus low trait anxious individuals. Cogn. Affect. Behav. Neurosci. 9, 323–334. 10.3758/CABN.9.3.323 19679767

[B25] Kiefer M. (2007). Top-down modulation of unconscious ‘automatic’ processes: a gating framework. Adv. Cogn. Psychol. 3, 289–306. 10.2478/v10053-008-0031-2 20517515 PMC2864982

[B26] Kiss M. Eimer M. (2008). ERPs reveal subliminal processing of fearful faces. Psychophysiology 45, 318–326. 10.1111/j.1469-8986.2007.00634.x 17995905 PMC2375009

[B27] Koivisto M. Kainulainen P. Revonsuo A. (2009). The relationship between awareness and attention: evidence from ERP responses. Neuropsychologia 47, 2891–2899. 10.1016/j.neuropsychologia.2009.06.016 19545577

[B28] Koivisto M. Revonsuo A. (2003). An ERP study of change detection, change blindness, and visual awareness. Psychophysiology 40, 423–429. 10.1111/1469-8986.00044 12946115

[B29] Koivisto M. Revonsuo A. (2007). Electrophysiological correlates of visual consciousness and selective attention. Neuroimage 18, 753–756. 10.1097/wnr.0b013e3280c143c8 17471060

[B30] Koivisto M. Revonsuo A. (2008). The role of selective attention in visual awareness of stimulus features: electrophysiological studies. Cogn. Affect. Behav. Neurosci. 8, 195–210. 10.3758/CABN.8.2.195 18589509

[B31] Koivisto M. Revonsuo A. (2010). Event-related brain potential correlates of visual awareness. Neurosci. Biobehav. Rev. 34, 922–934. 10.1016/j.neubiorev.2009.12.002 20005249

[B32] Lamy D. Salti M. Bar-Haim Y. (2008). Neural correlates of subjective awareness and unconscious processing: an ERP study. J. Cogn. Neurosci. 21, 1435–1446. 10.1162/jocn.2009.21064 18702582

[B33] LeDoux J. (2007). The amygdala. Curr. Biol. 17, R868–R874. 10.1016/j.cub.2007.08.005 17956742

[B34] Lehmann D. Skrandies W. (1980). Reference-free identification of components of checkerboard-evoked multichannel potential fields. Electroencephalogr. Clin. Neurophysiol. 48, 609–621. 10.1016/0013-4694(80)90419-8 6155251

[B35] Liddell B. J. Brown K. J. Kemp A. H. Barton M. J. Das P. Peduto A. (2005). A direct brainstem-amygdala-cortical ‘alarm’ system for subliminal signals of fear. Neuroimage 24, 235–243. 10.1016/j.neuroimage.2004.08.016 15588615

[B36] Liddell B. J. Williams L. M. Rathjen J. Shevrin H. Gordon E. (2004). A temporal dissociation of subliminal versus supraliminal fear perception: an event-related potential study. J. Cogn. Neurosci. 16, 479–486. 10.1162/089892904322926809 15072682

[B37] Lin Z. Murray S. O. (2014). Priming of awareness or how not to measure visual awareness. J. Vis. 14, 27–27. 10.1167/14.1.27 24474824

[B38] Lindquist K. A. Wager T. D. Kober H. Bliss-Moreau E. Barrett L. F. (2012). The brain basis of emotion: a meta-analytic review. Behav. Brain Sci. 35, 121–143. 10.1017/S0140525X11000446 22617651 PMC4329228

[B39] Macmillan N. A. Creelman C. D. (1991). Detection Theory: A User’s Guide. Cambridge: Cambridge University Press.

[B40] Maratos F. A. (2011). Temporal processing of emotional stimuli: the capture and release of attention by angry faces. Emotion 11, 1242–1247. 10.1037/a0024279 21942702

[B41] McCormick P. A. (1997). Orienting attention without awareness. J. Exp. Psychol. Hum. Percept. Perform. 23, 168–180. 10.1037/0096-1523.23.1.168 9157183

[B42] Mühlberger A. Wieser M. Herrmann M. Weyers P. Tröger C. Pauli P. (2009). Early cortical processing of natural and artificial emotional faces differs between lower and higher socially anxious persons. J. Neural. Transm. 116, 735–746. 10.1007/s00702-008-0108-6 18784899

[B43] Navajas J. Rey H. G. Quian Quiroga R. (2014). Perceptual and contextual awareness: methodological considerations in the search for the neural correlates of consciousness. Front. Psychol. 5:959. 10.3389/fpsyg.2014.00959 25221537 PMC4148639

[B44] Neumann O. (1984). “Automatic processing: a review of recent findings and a plea for an old theory,” in Cognition and Motor Processes, eds Prinz W. Sanders A. F. (Berlin Springer), 245–293.

[B45] Öhman A. Flykt A. Esteves F. (2001). Emotion drives attention: detecting the snake in the grass. J. Exp. Psychol. Gen. 130, 466–478. 10.1037/0096-3445.130.3.466 11561921

[B46] Öhman A. Flykt A. Lundqvist D. (2000). “Unconscious emotion: evolutionary perspectives, psychophysiological data and neuropsychological mechanisms,” in Cognitive Neuroscience of Emotion. Series in Affective Science, eds Lane R. D. R. Nadel L. Ahern G. L. (Oxford: Oxford University Press), 296–327.

[B47] Okon-Singer H. Tzelgov J. Henik A. (2007). Distinguishing between automaticity and attention in the processing of emotionally significant stimuli. Emotion 7, 147–157. 10.1037/1528-3542.7.1.147 17352570

[B48] Oldfield R. C. (1971). The assessment and analysis of handedness: the Edinburgh inventory. Neuropsychologia 9, 97–113. 10.1016/0028-3932(71)90067-4 5146491

[B49] Pegna A. J. Darque A. Berrut C. Khateb A. (2011). Early ERP modulation for task-irrelevant subliminal faces. Front. Psychol. 2:88. 10.3389/fpsyg.2011.00088 21687457 PMC3110345

[B50] Pegna A. J. Landis T. Khateb A. (2008). Electrophysiological evidence for early non-conscious processing of fearful facial expressions. Int. J. Psychophysiol. 70, 127–136. 10.1016/j.ijpsycho.2008.08.007 18804496

[B51] Pessoa L. Japee S. Ungerleider L. G. (2005a). Visual awareness and the detection of fearful faces. Emotion 5, 243–247. 10.1037/1528-3542.5.2.243 15982091

[B52] Pessoa L. Padmala S. Morland T. (2005b). Fate of unattended fearful faces in the amygdala is determined by both attentional resources and cognitive modulation. Neuroimage 28, 249–255. 10.1016/j.neuroimage.2005.05.048 15993624 PMC2427145

[B53] Pessoa L. Kastner S. Ungerleider L. G. (2002a). Attentional control of the processing of neutral and emotional stimuli. Cogn. Brain Res. 15, 31–45. 10.1016/S0926-6410(02)00214-8 12433381

[B54] Pessoa L. McKenna M. Gutierrez E. Ungerleider L. G. (2002b). Neural processing of emotional faces requires attention. Proc. Natl. Acad. Sci. U.S.A. 99, 11458–11463. 10.1073/pnas.172403899 12177449 PMC123278

[B55] Posner M. I. Snyder C. R. R. (1975). “Attention and cognitive control,” in Information Processing and Cognition: The Loyola Symposium, ed. Solso R. L. (Hillsdale: Lawrence Erlbaum Associates), 55–85.

[B56] Pourtois G. Grandjean D. Sander D. Vuilleumier P. (2004). Electrophysiological correlates of rapid spatial orienting towards fearful faces. Cereb. Cortex 14, 619–633. 10.1093/cercor/bhh023 15054077

[B57] Pourtois G. Schettino A. Vuilleumier P. (2013). Brain mechanisms for emotional influences on perception and attention: what is magic and what is not. Biol. Psychol. 92, 492–512. 10.1016/j.biopsycho.2012.02.007 22373657

[B58] Proverbio A. Del Zotto M. Zani A. (2010). Electrical neuroimaging evidence that spatial frequency-based selective attention affects V1 activity as early as 40–60 ms in humans. BMC Neurosci. 11:59. 10.1186/1471-2202-11-59 20459601 PMC2890012

[B59] Putman P. (2011). Resting state EEG delta-beta coherence in relation to anxiety, behavioral inhibition, and selective attentional processing of threatening stimuli. Int. J. Psychophysiol. 80, 63–68. 10.1016/j.ijpsycho.2011.01.011 21277914

[B60] Quek G. L. Finkbeiner M. (2013). Spatial and temporal attention modulate the early stages of face processing: behavioural evidence from a reaching paradigm. PLoS ONE 8:e57365. 10.1371/journal.pone.0057365 23468977 PMC3585364

[B61] Railo H. Revonsuo A. Koivisto M. (2015). Behavioral and electrophysiological evidence for fast emergence of visual consciousness. Neurosci. Conscious. 10.1093/nc/niv004 PMC6368270 30774982

[B62] Rossi V. Pourtois G. (2013). State-dependent attention modulation of human primary visual cortex: a high density ERP study. Neuroimage 60, 2365–2378. 10.1016/j.neuroimage.2012.02.007 22361168

[B63] Rutiku R. Martin M. Bachmann T. Aru J. (2015). Does the P300 reflect conscious perception or its consequences? Neuroscience 298, 180–189. 10.1016/j.neuroscience.2015.04.029 25907442

[B64] Shafto J. P. Pitts M. A. (2015). Neural signatures of conscious face perception in an inattentional blindness paradigm. J. Neurosci. 35, 10940–10948. 10.1523/JNEUROSCI.0145-15.2015 26245958 PMC6605277

[B65] Spielberger C. Gorsuch R. Lushene R. (1970). Manual for the State-Trait Anxiety Inventory. Palo Alto, CA: Consulting Psychologists Press.

[B66] Stolarova M. Keil A. Moratti S. (2006). Modulation of the C1 visual event-related component by conditioned stimuli: evidence for sensory plasticity in early affective perception. Cereb. Cortex 16, 876–887. 10.1093/cercor/bhj031 16151178

[B67] Tallon-Baudry C. (2012). On the neural mechanisms subserving consciousness and attention. Front. Psychol. 2:397. 10.3389/fpsyg.2011.00397 22291674 PMC3253412

[B68] Van den Bussche E. Van den Noortgate W. Reynvoet B. (2009). Mechanisms of masked priming: a meta-analysis. Psychol. Bull. 135, 452–477. 10.1037/a0015329 19379025

[B69] Vanlessen N. Rossi V. De Raedt R. Pourtois G. (2012). Positive emotion broadens attention focus through decreased position-specific spatial encoding in early visual cortex: evidence from ERPs. Cogn. Affect. Behav. Neurosci. 13, 60–79. 10.3758/s13415-012-0130-x 23090718

[B70] Vanni S. Revonsuo A. Saarinen J. Hari R. (1996). Visual awareness of objects correlates with activity of right occipital cortex. Neuroimage 8, 183–186. 10.1097/00001756-199612200-00037 9051777

[B71] Vuilleumier P. Armony J. L. Driver J. Dolan R. J. (2001). Effects of Attention and Emotion on Face Processing in the Human Brain: An Event-Related fMRI Study. Neuron 30, 829–841. 11430815 10.1016/s0896-6273(01)00328-2

[B72] Vuilleumier P. (2005). How brains beware: neural mechanisms of emotional attention. Trends Cogn. Sci. 9, 585–594. 10.1016/j.tics.2005.10.011 16289871

[B73] Wronka E. Walentowska W. (2011). Attention modulates emotional expression processing. Psychophysiology 48, 1047–1056. 10.1111/j.1469-8986.2011.01180.x 21332489

[B74] Yates A. Ashwin C. Fox E. (2010). Does emotion processing require attention? The effects of fear conditioning and perceptual load. Emotion 10, 822–830. 10.1037/a0020325 21058839 PMC3491873

[B75] Zani A. Proverbio A. (2012). Is that a belt or a snake? object attentional selection affects the early stages of visual sensory processing. Behav. Brain Funct. 8, 6. 10.1186/1744-9081-8-6 22300540 PMC3355026

[B76] Zani A. Proverbio A. M. (2009). Selective attention to spatial frequency gratings affects visual processing as early as 60 msec poststimulus. Percept. Mot. Skills 109, 140–158. 10.2466/pms.109.1.140-158 19831095

